# Can ChatGPT write better scientific titles? A comparative evaluation of human-written and AI-generated titles

**DOI:** 10.12688/f1000research.173647.1

**Published:** 2025-12-30

**Authors:** Paul Sebo, Bing Nie, Ting Wang

**Affiliations:** 1 University Institute for Primary Care, University of Geneva, Geneva, Switzerland; 2 Zhejiang Tongji Vocational College of Science and Technology, Hangzhou, Zhejiang, China; 3 School of Library and Information Management, Emporia State University, Emporia, Kansas, USA

**Keywords:** AI, artificial intelligence, authorship, ChatGPT, comparison, rater, reader perception, scientific title, scientific writing, title

## Abstract

**Background:**

Large language models (LLMs) such as GPT-4 are increasingly used in scientific writing, yet little is known about how AI-generated scientific titles are perceived by researchers in terms of quality.

**Objective:**

To compare the perceived accuracy, appeal, and overall preference for AI-generated versus human-written scientific titles.

**Methods:**

We conducted a blinded comparative study with 21 researchers from diverse academic backgrounds. A random sample of 50 original titles was selected from 10 high-impact general internal medicine journals. For each title, an alternative version was generated using GPT-4.0. Each rater evaluated 50 pairs of titles, each pair consisting of one original and one AI-generated version, without knowing the source of the titles or the purpose of the study. For each pair, raters independently assessed both titles on perceived accuracy and appeal, and indicated their overall preference. We analyzed accuracy/appeal using Wilcoxon signed-rank tests and negative binomial models, preferences using McNemar’s test and mixed-effects logistic regression, and inter-rater agreement with Gwet’s AC.

**Results:**

AI-generated titles received significantly higher ratings for both perceived accuracy (mean=7.9 vs. 6.7, *p-value *<0.001) and appeal (mean=7.1 vs. 6.7, *p-value *<0.001) than human-written titles. The odds of preferring an AI-generated title were 1.7 times higher ( *p-value *=0.001), with 61.8% of 1,049 paired judgments favoring the AI version. Inter-rater agreement was moderate to substantial (Gwet’s AC: 0.54–0.70).

**Conclusions:**

AI-generated titles can surpass human-written titles in perceived accuracy, appeal, and preference, suggesting that LLMs may enhance the effectiveness of scientific communication. These findings support the responsible integration of AI tools in research.

## Introduction

The title of a scientific article plays a critical role in academic communication. More than a simple label, it serves as the first point of contact between the research and its potential audience, potentially influencing whether the article is read, cited, or even submitted for peer review. Several studies have shown that titles affect readership and citation rates, ^1– 8^ an effect that may be especially pronounced in high-impact journals, where competition for visibility is intense. A well-crafted title must strike a balance between scientific accuracy and appeal, providing a succinct yet informative summary of the study’s main objective or findings, while simultaneously engaging the curiosity of readers. ^8– 14^

Crafting such titles is a complex task. Authors must condense their work into a limited number of words without compromising on clarity, scientific integrity, or appeal. The title must reflect the content of the study while remaining concise and readable. Moreover, researchers often face additional constraints such as journal-specific formatting rules, word limits, or stylistic preferences. ^13– 16^ In this context, the choice of words and tone can affect how a study is perceived and disseminated across the scientific community. For example, titles that use assertive or attention-grabbing language may be more memorable or appealing, yet they risk overstating the results or introducing bias in interpretation. ^17, 18^

Recent advancements in natural language processing (NLP) have opened new avenues in scientific writing. Large language models (LLMs) such as ChatGPT, developed by OpenAI, have demonstrated the ability to generate fluent, coherent, and contextually appropriate texts in response to user prompts. ^19– 29^ These tools are increasingly being adopted to assist with various writing tasks, including summarization, translation, and scientific manuscript generation. While preliminary evidence suggests that LLMs can support academic writing tasks, their potential role in title generation remains largely unexplored. ^30, 31^

Chen and Eger (2023) assessed the performance of transformer-based models—including ChatGPT—in generating scientific titles from abstracts in the domains of NLP and machine learning. ^30^ Their study focused on stylistic aspects such as humor and novelty, and introduced the first large-scale dataset of humorous scientific titles. Although certain models (e.g., BARTxsum) produced titles approaching human-level quality, effectively capturing authentic humor remained a notable challenge. Rehman et al. (2024) used multiple pre-trained language models to generate titles for biomedical research articles and compared them to human-written titles using standard textual similarity metrics such as ROUGE, BLEU, and METEOR. ^31^ The AI-generated titles showed high lexical similarity with human titles, suggesting that these models can replicate conventional title structures. However, the study relied exclusively on automated metrics, without assessing how readers actually perceive these titles in terms of accuracy, appeal, or credibility. Moreover, the articles used in their study were from the post-2020 era, raising the possibility that human-written titles may themselves have been influenced by AI-assisted tools. As a result, it remains unclear whether LLMs like ChatGPT can independently produce high-quality scientific titles that are preferred by human readers.

To address this gap, the present study was designed to evaluate whether ChatGPT-4.0 can generate titles that are perceived as accurate, appealing, and overall preferable compared to those written by human authors. Our study is unique in three main respects. First, it uses articles from a period before AI tools existed, ensuring that the original titles are purely human-authored. Second, it evaluates the quality of titles using human perceptions (rather than automated similarity metrics) on key dimensions of interest to readers. Third, it uses ChatGPT-4.0, one of the most advanced publicly available LLMs to date, as a title-generation tool in a zero-shot setting, reflecting its potential use by researchers without engineering expertise. We hypothesized that titles generated by ChatGPT would be perceived as more accurate and appealing than those written by humans, and potentially preferred overall.

## Methods

### Study objective and design

This study aimed to evaluate the capacity of ChatGPT-4.0 to produce scientific article titles that are accurate, appealing, and preferred by readers. We compared AI-generated titles with original human-written titles drawn from high-impact journals in general internal medicine. Our objective was to assess whether ChatGPT could match or surpass human authors in crafting titles that effectively reflect the content of the article and attract readers’ interest. To this end, we conducted a cross-sectional survey in which independent academic raters evaluated paired titles for each of fifty scientific abstracts. Each abstract was presented with two titles, one written by a human, the other generated by ChatGPT, in randomized order to avoid bias.

### Journal and article selection

We first identified the ten general internal medicine journals with the highest impact factors in the 2023 Journal Citation Reports (JCR). To ensure consistency and relevance across journals, only those fulfilling all of the following criteria were eligible: they had to regularly publish original research and/or systematic reviews; they had to use structured abstracts for both types of articles; and they had to have been in continuous publication since at least January 2000. The year 2000 was deliberately chosen as the target publication period because it predates the availability of generative AI tools, eliminating any possibility that the original titles were AI-assisted. Based on these criteria, the following journals were selected: *The Lancet* (IF 98.4) *, The New England Journal of Medicine* (IF 96.3) *, The BMJ* (IF 93.7) *, JAMA* (IF 63.5) *, Archives of Internal Medicine* (IF 22.3) *, Annals of Internal Medicine* (IF 19.6) *, CMAJ* (IF 12.9) *, Journal of Travel Medicine* (IF 9.1) *, Journal of Internal Medicine* (IF 9.0) *, and Mayo Clinic Proceedings* (IF 6.9).

From each eligible journal, we randomly selected five articles published between January 1 and December 31, 2000. These articles were either original research studies or systematic reviews. This sampling strategy resulted in a total of fifty abstracts, each with a corresponding human-written title.

### AI-based title generation procedure

To generate alternative titles, we used the ChatGPT-4.0 model developed by OpenAI, which represents one of the most advanced publicly available LLMs at the time of the study. For each abstract, we initiated a new chat session with the model. This was done intentionally to eliminate contextual memory carryover and ensure that each title was generated independently of the others.

In each new session, the following standardized prompt was submitted: “ *Write a title for this scientific article based on the abstract below*”. Immediately after entering the prompt, we pasted the full abstract of the selected article. The AI-generated title that resulted from this process was recorded verbatim and was not edited, reformulated, or shortened in any way by the researchers, except for standardizing capitalization: words were converted to lowercase when uppercase was not required (e.g., unless referring to names, countries, or other proper nouns). This step was repeated for all fifty abstracts, yielding fifty unique AI-generated titles. The human-written and ChatGPT-generated titles are presented in the Supplementary Material.

### Pairing and randomization of titles

Each abstract was thus associated with two titles: one written by the original human authors and the other generated by ChatGPT-4.0. For evaluation purposes, the two titles were assigned randomized positions as either “Title A” or “Title B” using a simple randomization algorithm. This random order was intended to prevent raters from identifying which title had been written by a human and which by an AI, thereby minimizing bias during the evaluation process.

### Questionnaire development and rating criteria

A structured evaluation questionnaire was developed to assess rater perceptions of the two titles accompanying each abstract. The survey presented all fifty abstracts, each introduced by two titles in randomized order (Title A and Title B), followed by the abstract itself. Each rater was asked to assess each title separately on two dimensions: first, how well the title represented the content of the abstract, and second, how much the title made them want to read the abstract or the full article.

These two dimensions (i.e., perceived accuracy and appeal) were each rated using an ordinal scale ranging from 0 to 10. On this scale, a rating of 0 indicated an extremely negative judgment (e.g., not accurate or not appealing at all), a rating of 5 reflected a neutral or moderate assessment, and a rating of 10 indicated a highly positive evaluation (e.g., perfectly accurate or extremely appealing).

After rating both titles on these two aspects, the raters were also asked to indicate which of the two titles they preferred overall, choosing either “Title A” or “Title B” for each abstract. The questionnaire and rating form are available in the Supplementary Material.

### Rater recruitment and blinding

Twenty-one raters participated in the evaluation phase of the study. All were researchers who had authored at least one peer-reviewed academic publication. Eleven of these raters were recruited and contacted by one co-author (BN), and the remaining ten by another (PS), to ensure balanced recruitment. All participants provided informed consent in written electronic form (email agreement and completion of the questionnaire).

To avoid bias and maintain ecological validity, raters were not informed that one of the two titles had been generated by AI. They were simply told that the study aimed to examine how different formulations of article titles affect readers’ perceptions. No specific mention was made of ChatGPT or AI-based generation to preserve the authenticity of the evaluations.

### Data collection timeline

The process of generating AI-based titles was completed in May 2025. The rating process, during which the twenty recruited raters completed the questionnaire, was conducted throughout June 2025. All ratings were submitted electronically and compiled in a central database for further statistical analysis.

### Ethics and consent

This study did not require ethics committee approval under Swiss law, as no personal health data were collected (Human Research Act, HRA, art.2). All participants were adult researchers, informed about the study’s purpose (evaluating perceptions of different title formulations), voluntary participation, and anonymized handling of responses. To minimize bias, they were not told that one of the titles was AI-generated. Written informed consent was obtained via email agreement and completion of the questionnaire.

### Statistical analysis

For each title, we calculated the mean (standard deviation, SD) and median (interquartile range, IQR) of rater scores for perceived accuracy and appeal. To compare ratings between human-written and AI-generated titles, we used the Wilcoxon signed-rank test for paired data, as the ratings were ordinal and not normally distributed. For title preferences, we calculated the proportion of times each title was selected. Differences in preference proportions were tested using McNemar’s test, which is appropriate for paired categorical data.

In addition to these non-parametric tests, we conducted multilevel regression analyses to quantify effect sizes. Negative binomial regression models clustered by rater ID (for overdispersed counts) were used to compare rating counts for perceived accuracy and appeal, yielding incidence rate ratios (IRRs). A mixed-effects logistic regression model, also clustered by rater ID, was used to assess the odds of selecting an AI-generated title over a human-written one, yielding an odds ratio (OR) for preference.

To assess inter-rater agreement, we computed two measures separately for AI-generated and human-written titles: percent agreement and Gwet’s agreement coefficient (AC), using quadratic weights to account for the ordinal nature of the 1–10 rating scale. ^32– 34^ Agreement levels were computed across the 21 raters and stratified by rating dimension (perceived accuracy and appeal). The weighted analysis assigns partial credit for near agreement, making it more appropriate for ordinal data. We interpreted Gwet’s AC using the classification proposed by Landis and Koch (1977): values <0.00 indicate poor agreement, 0.00–0.20 slight, 0.21–0.40 fair, 0.41–0.60 moderate, 0.61–0.80 substantial, and 0.81–1.00 almost perfect agreement. ^35^

We did not perform subgroup analyses based on rater characteristics, as the limited number of raters (N = 21) would not have allowed for statistically meaningful comparisons. All analyses were conducted using Stata version 15.1 (StataCorp, College Station, TX, USA). A two-sided p-value < 0.05 was considered statistically significant.

## Results

### Rater characteristics

The main characteristics of the 21 raters who participated in the study are presented in Table 1. Twelve were women and nine were men. Twelve were under 40 years of age, eight were between 40 and 60 years, and one was over 60 years old. The raters were primarily from China (n = 11) and Switzerland (n = 8), with one rater each from the United States and France. They had diverse academic and professional backgrounds. Among them, five specialized in library and information science, and seven in general internal medicine.

**Table 1.** Characteristics of the 21 raters who evaluated 50 scientific titles from 10 high-impact general internal medicine journals.

Rater ID	Initials	Gender	Age group	Work city	Work country	Discipline
1	Y.W.	Male	<40	Qingdao	China	General internal medicine
2	YC.B	Female	<40	Suzhou	China	Bioinformatics
3	MJ.G.	Female	40-60	Hangzhou	China	Library and information science
4	B.Z.	Female	40-60	Hangzhou	China	Arts
5	RD.J.	Male	40-60	Hangzhou	China	International Chinese education
6	BF.S.	Female	<40	Hangzhou	China	Library and information science
7	CQ.W.	Female	<40	Hangzhou	China	Political economics
8	HS.X.	Female	<40	Guangzhou	China	Psychiatry
9	Y.L.	Male	<40	Guangzhou	China	Psychiatry
10	Y.W.	Female	<40	Guangzhou	China	Psychiatry
11	B.N.	Female	<40	Hangzhou	China	Library and information science
12	S.DL.	Male	40-60	Geneva	Switzerland	General internal medicine
13	B.T.	Male	40-60	Lyon	France	General internal medicine
14	A.M.	Male	<40	Geneva	Switzerland	Library and information science
15	M.B.	Male	40-60	Geneva	Switzerland	General internal medicine and angiology
16	N.P.	Male	40-60	Geneva	Switzerland	General internal medicine
17	C.K.	Male	>60	Geneva	Switzerland	Anaesthesia
18	N.W.	Female	<40	Zurich	Switzerland	General internal medicine and cardiology
19	L.M.	Female	<40	Geneva	Switzerland	General internal medicine
20	E.D.	Female	40-60	Geneva	Switzerland	Public health
21	T.W.	Female	<40	Emporia	USA	Library and information science

### Perceived accuracy and appeal ratings

**Figure 1.**
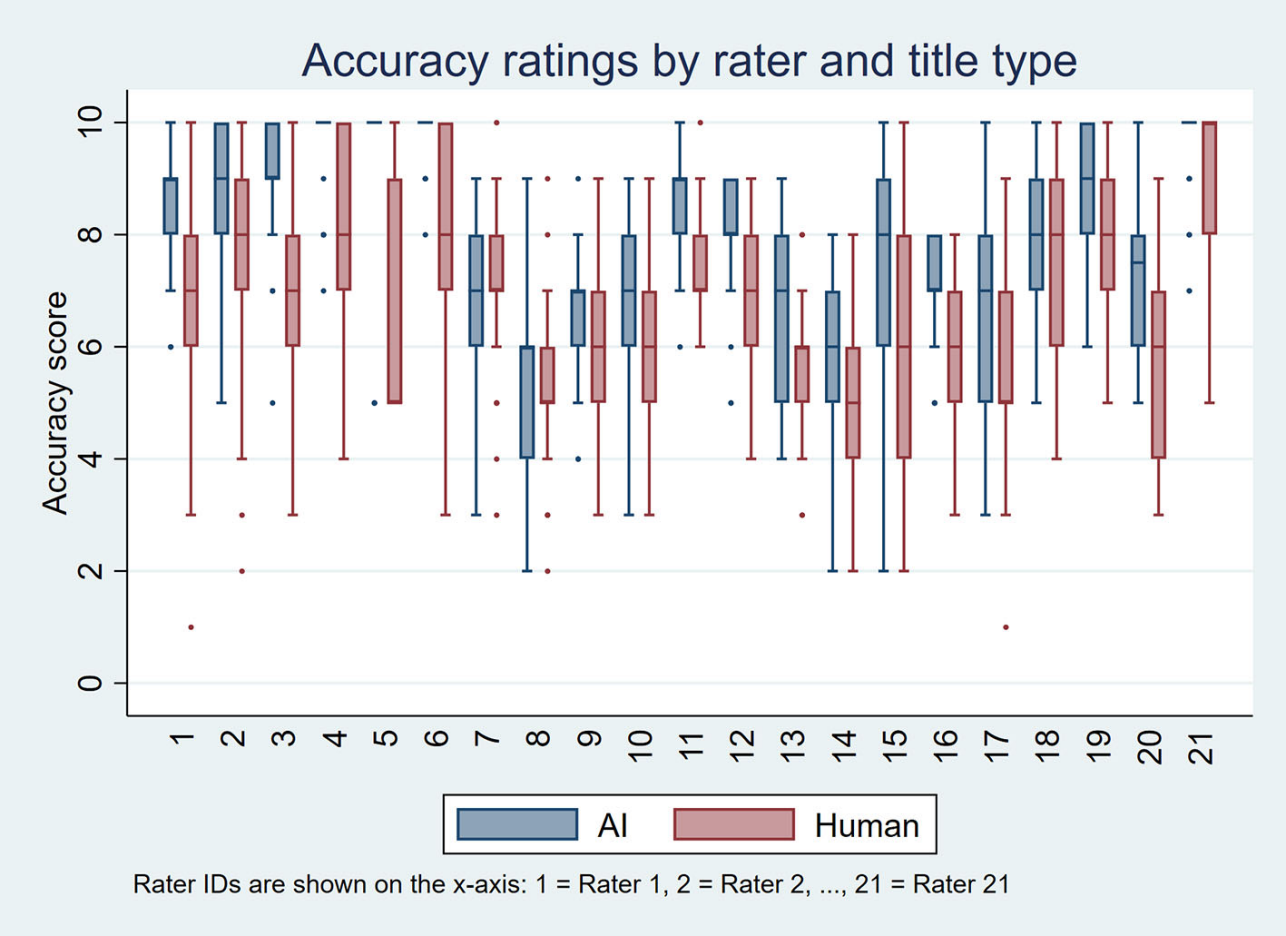
Boxplots showing perceived accuracy ratings for AI-generated and human-written titles for each of the 21 raters, based on 50 scientific titles from 10 high-impact general internal medicine journals.

**Figure 2.**
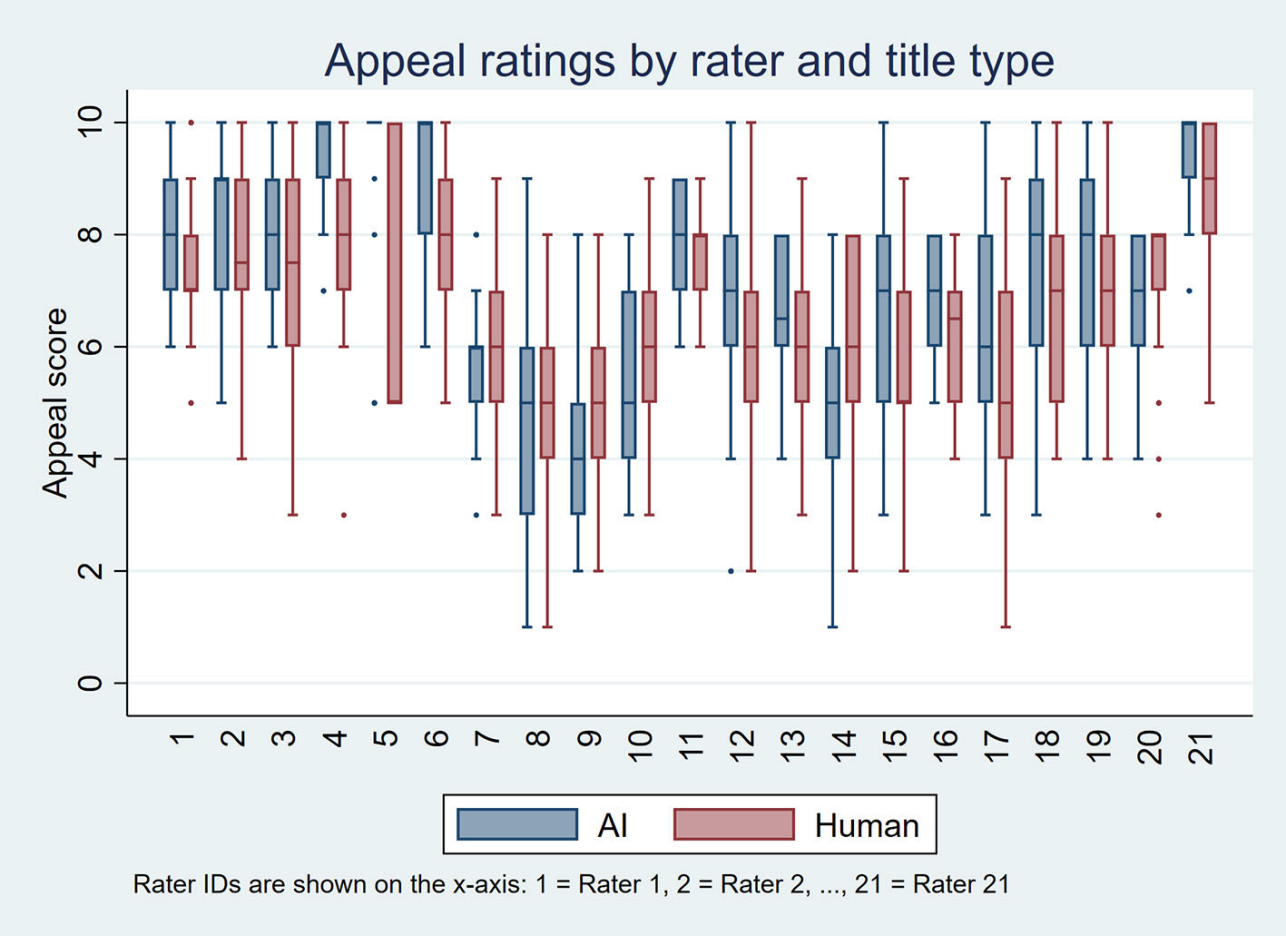
Boxplots showing appeal ratings for AI-generated and human-written titles for each of the 21 raters, based on 50 scientific titles from 10 high-impact general internal medicine journals.

Table 2 presents the median, IQR, and minimum–maximum values of rater scores for perceived accuracy and appeal, stratified by title type (AI-generated vs. human-written) and by individual rater. Figures 1 and 2 display these distributions using boxplots, one per rater, for perceived accuracy and appeal, respectively. Overall, AI-generated titles received more favorable ratings. For perceived accuracy, 18 raters rated AI-generated titles higher than human-written titles, three gave equal ratings, and none rated AI-generated titles lower. For appeal, 12 raters rated AI-generated titles higher, five gave equal ratings, and four preferred human-written titles.

**Table 2.** Summary of rater scores (median, IQR, min, max) for perceived accuracy and appeal by title type and rater ID, based on 50 scientific titles from 10 general internal medicine journals.

Rater ID	Title type	Dimension	Median	P25 ^1^	P75 ^1^	Min	Max
1	AI	accuracy	9	8	9	6	10
1	AI	appeal	8	7	9	6	10
1	Human	accuracy	7	6	8	1	10
1	Human	appeal	7	7	8	5	10
2	AI	accuracy	9	8	10	5	10
2	AI	appeal	9	7	9	5	10
2	Human	accuracy	8	7	9	2	10
2	Human	appeal	8	7	9	4	10
3	AI	accuracy	9	9	10	5	10
3	AI	appeal	8	7	9	6	10
3	Human	accuracy	7	6	8	3	10
3	Human	appeal	8	6	9	3	10
4	AI	accuracy	10	10	10	7	10
4	AI	appeal	10	9	10	7	10
4	Human	accuracy	8	7	10	4	10
4	Human	appeal	8	7	9	3	10
5	AI	accuracy	10	10	10	5	10
5	AI	appeal	10	10	10	5	10
5	Human	accuracy	5	5	9	5	10
5	Human	appeal	5	5	10	5	10
6	AI	accuracy	10	10	10	8	10
6	AI	appeal	10	8	10	6	10
6	Human	accuracy	8	7	10	3	10
6	Human	appeal	8	7	9	5	10
7	AI	accuracy	7	6	8	3	9
7	AI	appeal	6	5	6	3	8
7	Human	accuracy	7	7	8	3	10
7	Human	appeal	6	5	7	3	9
8	AI	accuracy	6	4	6	2	9
8	AI	appeal	5	3	6	1	9
8	Human	accuracy	5	5	6	2	9
8	Human	appeal	5	4	6	1	8
9	AI	accuracy	7	6	7	4	9
9	AI	appeal	4	3	5	2	8
9	Human	accuracy	6	5	7	3	9
9	Human	appeal	5	4	6	2	8
10	AI	accuracy	7	6	8	3	9
10	AI	appeal	5	4	7	3	8
10	Human	accuracy	6	5	7	3	9
10	Human	appeal	6	5	7	3	9
11	AI	accuracy	9	8	9	6	10
11	AI	appeal	8	7	9	6	9
11	Human	accuracy	7	7	8	6	10
11	Human	appeal	8	7	8	6	9
12	AI	accuracy	8	8	9	5	9
12	AI	appeal	7	6	8	2	10
12	Human	accuracy	7	6	8	4	9
12	Human	appeal	6	5	7	2	10
13	AI	accuracy	7	5	8	4	9
13	AI	appeal	7	6	8	4	8
13	Human	accuracy	6	5	6	3	8
13	Human	appeal	6	5	7	3	9
14	AI	accuracy	6	5	7	2	8
14	AI	appeal	5	4	6	1	8
14	Human	accuracy	5	4	6	2	8
14	Human	appeal	6	5	8	2	8
15	AI	accuracy	8	6	9	2	10
15	AI	appeal	7	5	8	3	10
15	Human	accuracy	6	4	8	2	10
15	Human	appeal	5	5	7	2	9
16	AI	accuracy	7	7	8	5	8
16	AI	appeal	7	6	8	5	8
16	Human	accuracy	6	5	7	3	8
16	Human	appeal	7	5	7	4	8
17	AI	accuracy	7	5	8	3	10
17	AI	appeal	6	5	8	3	10
17	Human	accuracy	5	5	7	1	9
17	Human	appeal	5	4	7	1	9
18	AI	accuracy	8	7	9	5	10
18	AI	appeal	8	6	9	3	10
18	Human	accuracy	8	6	9	4	10
18	Human	appeal	7	5	8	4	10
19	AI	accuracy	9	8	10	6	10
19	AI	appeal	8	6	9	4	10
19	Human	accuracy	8	7	9	5	10
19	Human	appeal	7	6	8	4	10
20	AI	accuracy	8	6	8	5	10
20	AI	appeal	7	6	8	4	8
20	Human	accuracy	6	4	7	3	9
20	Human	appeal	8	7	8	3	8
21	AI	accuracy	10	10	10	7	10
21	AI	appeal	10	9	10	7	10
21	Human	accuracy	10	8	10	5	10
21	Human	appeal	9	8	10	5	10

^1^ P25: 25th percentile. P75: 75th percentile. Each rater evaluated both AI-generated and human-written titles for perceived accuracy and appeal. Ratings range from 1 to 10.

**Figure 3.**
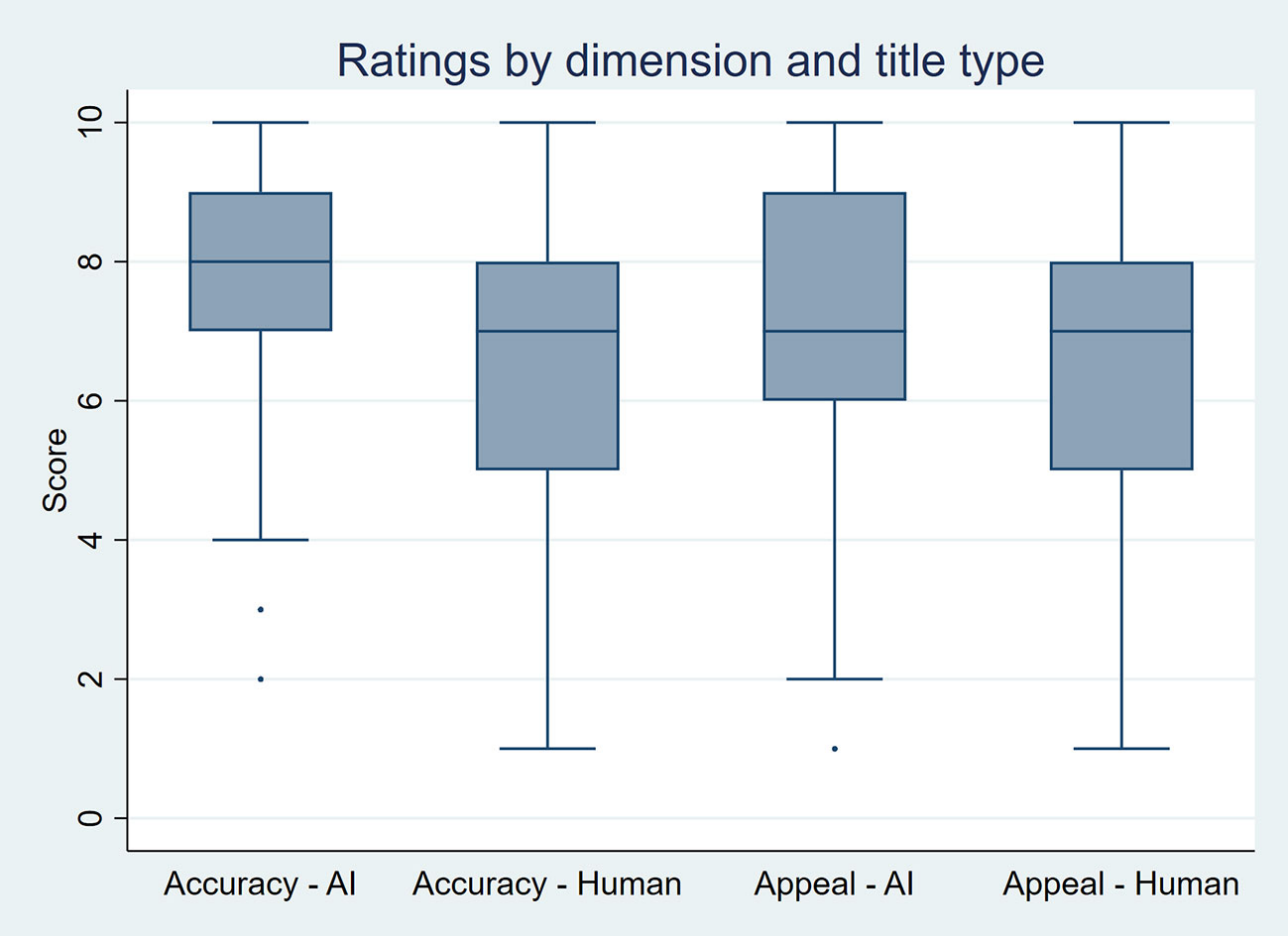
Boxplots showing perceived accuracy and appeal ratings for AI-generated and human-written titles by 21 raters, based on 50 scientific titles from 10 high-impact general internal medicine journals.

As summarized in Table 3 and visualized in Figure 3, AI-generated titles received significantly higher scores. For perceived accuracy, the mean score was 7.9 for AI-generated titles compared to 6.7 for human-written titles, with a median of 8 versus 7 (p-value < 0.001). For appeal, the mean score was 7.1 for AI-generated titles versus 6.7 for human-written titles, with a median of 7 for both (p-value < 0.001). In terms of incidence rate ratios (IRRs), ratings for AI-generated titles were 1.17 times higher for perceived accuracy and 1.06 times higher for appeal compared to human-written titles (p-value < 0.001 and p-value = 0.02, respectively).

**Table 3.** Perceived accuracy and appeal ratings, and title preferences, by title type, based on 4,196 ratings from 21 raters who evaluated 50 scientific titles from 10 high-impact general internal medicine journals.

	Number of ratings/total	Mean (SD)	Median (IQR)	Min-max	N (%)	p-value	OR or IRR (95% CI)	p-value
Perceived accuracy						<0.001 ^1^		<0.001 ^3^
AI-generated title	1049/1050	7.9 (1.8)	8 (7-9)	2-10			1.17 (1.13-1.22)	
Human title	1049/1050	6.7 (1.9)	7 (5-8)	1-10			1 (ref )	
Appeal						<0.001 ^1^		0.02 ^3^
AI-generated title	1049/1050	7.1 (2.1)	7 (6-9)	1-10			1.06 (1.01-1.12)	
Human title	1049/1050	6.7 (1.8)	7 (5-8)	1-10			1 (ref )	
Preference						<0.001 ^2^		0.001 ^4^
AI-generated title	1049/1050				648 (61.8)		1.69 (1.25-2.27)	
Human title	1049/1050				401 (38.2)		1 (ref )	

^1^ *Wilcoxon signed-rank tests (paired, one rating per title per rater).*

^2^ *McNemar’s test (paired binary preferences, one per title per rater).*

^3^ *Incidence rate ratios (IRRs) and p-values are from negative binomial regression models clustered by rater ID. The ratings for AI-generated titles were 1.17 times higher for accuracy and 1.06 times higher for appeal, compared to human-written titles.*

^4^ *Odds ratio (OR) and p-value are from a mixed-effects logistic regression model clustered by rater ID. The odds of preferring an AI-generated title were 1.69 times higher than for a human-written title.*

### Title preferences

**Figure 4.**
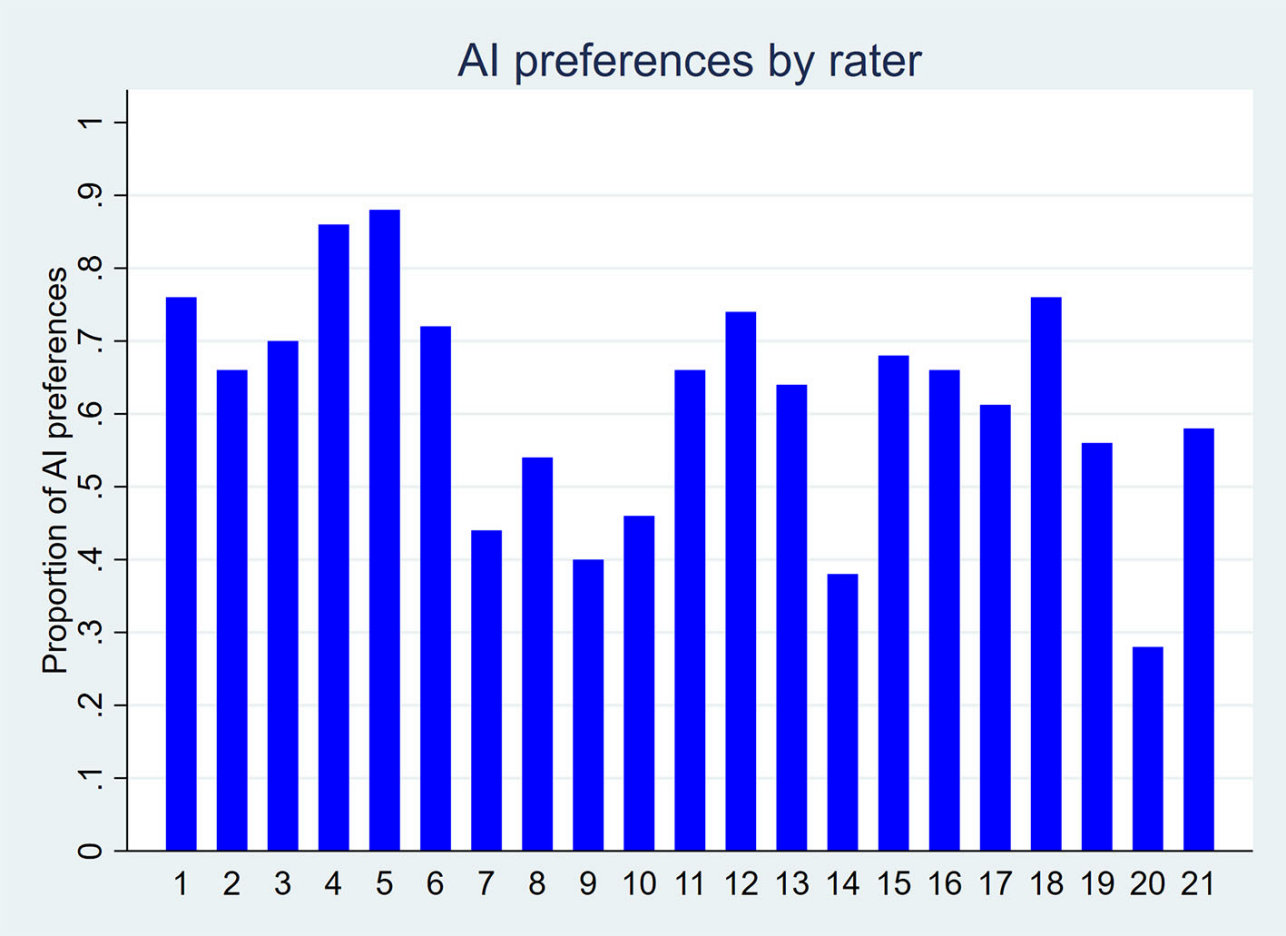
Proportion of AI-generated title preferences for each of the 21 raters, based on 50 scientific titles from 10 high-impact general internal medicine journals.

Overall preferences also favored AI-generated titles. As shown in Figure 4, 16 out of 21 raters preferred AI-generated titles, while five preferred human-written ones. Among the 1,049 pairwise preference judgments (out of a possible 1,050; one missing value), 61.8% favored the AI-generated title and 38.2% favored the human-written title (p-value < 0.001; Table 3). The odds of preferring an AI-generated title were 1.69 times higher than those of preferring a human-written title (p-value = 0.001).

### Inter-rater agreement

Table 4 presents inter-rater agreement measures by title type. Percent agreement ranged from 88.9% to 92.5%, while Gwet’s ACs, calculated using quadratic weights for ordinal scales, ranged from 0.54 to 0.70. These values indicate moderate to substantial agreement according to the benchmark scale proposed by Landis and Koch (1977). ^35^

**Table 4.** Inter-rater agreement on perceived accuracy and appeal ratings, by title type, based on 4,196 ordinal ratings (scale 1–10) from 21 raters who evaluated 50 scientific titles from 10 high-impact general internal medicine journals, using quadratic weights.

Dimension	Title type	Percent agreement (95% CI)	p-value	Gwet’s agreement coefficient (95% CI)	p-value
Accuracy	AI-generated	0.8965 (0.8876-0.9055)	<0.001	0.6141 (0.5741-0.6541)	<0.001
Accuracy	Human-written	0.9254 (0.9190-0.9318)	<0.001	0.7029 (0.6715-0.7343)	<0.001
Appeal	AI-generated	0.8890 (0.8809-0.8971)	<0.001	0.5378 (0.4964-0.5793)	<0.001
Appeal	Human-written	0.9198 (0.9123-0.9274)	<0.001	0.6845 (0.6466-0.7223)	<0.001

## Discussion

### Summary of key findings

This study evaluated how 21 raters assessed the perceived accuracy, appeal, and overall preference of 50 scientific titles, comparing AI-generated and human-written versions. AI-generated titles received significantly higher ratings for both perceived accuracy and appeal, with most raters favoring them over human-written alternatives. In total, 61.8% of preference judgments were in favor of AI-generated titles, and inter-rater agreement ranged from moderate to substantial.

### Comparison with literature

Our findings are consistent with a growing body of literature suggesting that LLMs such as GPT-4.0 can generate high-quality scientific text that is often indistinguishable from human-written content. ^20, 36– 42^ Our results go beyond prior work by focusing specifically on titles, a concise yet crucial form of scientific communication. Unlike abstracts or full texts, titles must strike a balance between informativeness, clarity, and appeal in a highly constrained format. While some recent studies have explored AI-generated titles, they have either emphasized stylistic aspects such as humor and novelty in technical fields or evaluated output using only automated similarity metrics, without considering how human readers perceive title quality. ^30, 31^ The fact that AI-generated titles scored higher on both perceived accuracy and appeal challenges assumptions that LLMs lack the nuance or domain expertise to outperform human authors in such a delicate task. This suggests that LLMs may be particularly well suited for short-form scientific writing, where lexical clarity and stylistic optimization matter more than in-depth reasoning.

Importantly, our study focused exclusively on articles from high-impact general internal medicine journals, where title quality is expected to be particularly high due to rigorous editorial and peer-review processes. If AI-generated titles can outperform those published in such venues, the gap may be even greater for titles in lower-tier journals, where writing quality is more variable. Future research should investigate whether similar results hold across different fields, disciplines, and levels of journal prestige.

Collectively, our study complements and extends previous research by offering a detailed, comparative analysis of AI vs. human performance in scientific titling, a topic that has received relatively little empirical attention but has major implications for academic publishing practices.

### Implications for practice and research

From a practical standpoint, the finding that AI-generated titles are rated more highly than human-written ones suggests that LLMs could be reliably used to assist researchers in generating or refining article titles. Given that titles play a key role in shaping reader perceptions, citation rates, and online discoverability, tools that enhance title quality could have a direct impact on dissemination and academic impact. In particular, researchers with limited writing experience or for whom English is not a first language might benefit from LLM-based titling tools to improve clarity and reader engagement.

The observed preferences imply that AI-generated suggestions may outperform human intuition in specific aspects of scientific writing, such as title generation. This raises the possibility of integrating AI assistance more formally into journal workflows, for example through automated title suggestions during the submission process or editorial review. While this would require careful oversight, our data indicate that such tools would not compromise, and may even enhance, perceived quality.

However, the integration of AI into scholarly communication also raises critical ethical questions. ^29, 43– 46^ These concerns echo ongoing debates about the role of LLMs in scientific authorship and the boundaries of acceptable assistance. Our findings underline the importance of maintaining transparent authorship practices and labeling AI contributions in scientific writing, even if such tools are only used to generate the title of the article. In addition, the widespread application of AI in generating titles may lead to homogenization in academic writing, resulting in titles that tend to fall within a narrow stylistic range and suppress the diversity, creativity, and uniqueness of the disciplines.

From a research perspective, our study opens several avenues for further investigation. One important direction is to test the generalizability of these findings across disciplines, languages, and types of scientific content. It is possible that preferences for AI-generated titles vary depending on disciplinary norms or journal styles. In addition, future work could examine how title preferences correlate with actual article impact, such as downloads, citations, or Altmetric scores, to determine whether rater judgments align with broader readership behavior. Another key area for future research is to understand the mechanisms behind rater preferences. For example, are AI-generated titles preferred because of greater lexical simplicity, more direct structure, or the avoidance of technical jargon? Applying NLP tools to analyze linguistic features could shed light on what drives these preferences and help refine AI title generation even further. Lastly, as LLMs continue to evolve, longitudinal studies will be needed to assess how perceptions of AI-generated text change over time and whether improvements in model quality lead to higher standards or greater acceptance.

### Limitations

This study has several limitations that should be acknowledged. First, although the use of articles from the year 2000 ensured that original titles were free from AI influence, it also introduces a potential temporal bias. Scientific writing conventions and stylistic preferences may have evolved over the past two decades, and what was considered an effective title in 2000 may differ from current standards. Second, although we recruited raters with relevant academic experience, the sample size (N = 21) remains relatively small, and their subjective preferences may not fully represent broader readership or editorial perspectives. Third, while the zero-shot setting of ChatGPT-4.0 reflects real-world usage by non-expert users, it may not capture the full potential of LLMs when used with prompt optimization or human-in-the-loop refinement. Additionally, the evaluation focused on only two dimensions (i.e., perceived accuracy and appeal) along with an overall preference rating. Other important aspects of scientific titles, such as clarity, informativeness, tone, and appropriateness for indexing or search engine optimization, were not explicitly assessed. Lastly, the study did not include domain experts for each article’s specific topic area, which may have influenced the ability of raters to judge how well a title reflected the article’s nuanced content.

Future research could expand upon this work by including more diverse raters, evaluating newer articles, testing various prompting strategies, and incorporating additional dimensions of title quality. Despite these limitations, our findings provide valuable insights into the potential of LLMs to assist in academic title generation and highlight the subjective nature of title preferences.

## Conclusion

This study provides empirical evidence that AI-generated scientific titles can outperform human-written titles in perceived accuracy, appeal, and overall preference. On average, AI-generated titles received higher ratings and were preferred more often, with moderate to substantial agreement between raters. These findings suggest that LLMs like GPT-4.0 are not only capable of producing linguistically fluent content but may also enhance key aspects of scientific communication. As AI tools become more integrated into the research and publishing process, there is a timely opportunity to harness their strengths while remaining attentive to ethical considerations, disciplinary norms, and the evolving expectations of scientific readers.

## Ethical approval

Since this study did not involve the collection of personal health-related data it did not require ethical review, according to current Swiss law (Human Research Act, HRA, art.2).

## Data Availability

Open Science Framework: *Can ChatGPT write better scientific titles? A comparative evaluation of human-written and AI-generated titles.* https://doi.org/10.17605/OSF.IO/NF8ZR ^47^ This project contains the following underlying data:
•
**
title_data_SM.xlsx** – raw accuracy and appeal ratings for each title (AI vs. human) evaluated by 21 raters. Open Science Framework: *Can ChatGPT write better scientific titles? A comparative evaluation of human-written and AI-generated titles.* https://doi.org/10.17605/OSF.IO/NF8ZR ^47^ This project contains the following extended data:
•
**
title_chatgpt_SM.docx** – table of the 50 original human-written titles and the 50 ChatGPT-generated titles, and the questionnaire used for rating accuracy, appeal, and preference. Data are available under the terms of the Creative Commons Attribution licence (CC-BY 4.0).

## References

[1] Paiva CE Lima JP d SN Paiva BSR : Articles with short titles describing the results are cited more often. *Clinics.* 2012;67(5):509–513. 10.6061/clinics/2012(05)17 22666797 PMC3351256

[2] Jacques TS Sebire NJ : The Impact of Article Titles on Citation Hits: An Analysis of General and Specialist Medical Journals. *JRSM Short Rep.* 2010;1(1):1–5. 10.1258/shorts.2009.100020 21103094 PMC2984326

[3] Sagi I Yechiam E : Amusing titles in scientific journals and article citation. *J. Inf. Sci.* 2008;34(5):680–687. 10.1177/0165551507086261

[4] Jamali HR Nikzad M : Article title type and its relation with the number of downloads and citations. *Scientometrics.* 2011;88(2):653–661. 10.1007/s11192-011-0412-z

[5] Letchford A Moat HS Preis T : The advantage of short paper titles. *R. Soc. Open Sci.* 2015;2(8):150266. 10.1098/rsos.150266 26361556 PMC4555861

[6] Rostami F Mohammadpoorasl A Hajizadeh M : The effect of characteristics of title on citation rates of articles. *Scientometrics.* 2014;98(3):2007–2010. 10.1007/s11192-013-1118-1

[7] Subotic S Mukherjee B : Short and amusing: The relationship between title characteristics, downloads, and citations in psychology articles. *J. Inf. Sci.* 2014;40(1):115–124. 10.1177/0165551513511393

[8] Gichuki D : The Role of Scientific Titles in Shaping Research Visibility and Citations. *Int. J. Res. Innov. Appl. Sci.* 2024;IX(X):511–515. 10.51584/IJRIAS.2024.910046

[9] Lewison G Hartley J : What’s in a title? Numbers of words and the presence of colons. *Scientometrics.* 2005;63(2):341–356. 10.1007/s11192-005-0216-0

[10] Chamorro-Padial J Rodríguez-Sánchez R : The relevance of title, abstract, and keywords for scientific paper quality and potential impact. *Multimed. Tools Appl.* 2023;82(15):23075–23090. 10.1007/s11042-023-14451-9

[11] Tips for constructing an effective title. *Nat. Biomed. Eng.* 2022;6(2):105–105. 10.1038/s41551-022-00858-6 35190676

[12] Pottier P Lagisz M Burke S : Title, abstract and keywords: a practical guide to maximize the visibility and impact of academic papers. *Proc. R. Soc. B Biol. Sci.* 2027;291(291):20241222. 10.1098/rspb.2024.1222 39079668 PMC11288685

[13] Tullu MS : Writing the title and abstract for a research paper: Being concise, precise, and meticulous is the key. *Saudi J. Anaesth.* 2019;13(Suppl 1):S12–S17. 10.4103/sja.SJA_685_18 30930712 PMC6398294

[14] Kretchmer: Title Optimization for Peer-Reviewed Journals: How to increase Citations. *San Francisco Edit. * June 25, 2024. Accessed June 7, 2025. Reference Source

[15] Medical Writing 4th Edition: Cambridge University Press & Assessment. Accessed June 7, 2025. Reference Source

[16] Hartley J : Current findings from research on structured abstracts. *J. Med. Libr. Assoc.* 2004;92(3):368–371. 15243644 PMC442180

[17] Vinkers CH Tijdink JK Otte WM : Use of positive and negative words in scientific PubMed abstracts between 1974 and 2014: retrospective analysis. *BMJ.* 2015;351:h6467. 10.1136/bmj.h6467 26668206 PMC4677695

[18] Yavchitz A Boutron I Bafeta A : Misrepresentation of Randomized Controlled Trials in Press Releases and News Coverage: A Cohort Study. *PLoS Med.* 2012;9(9):e1001308. 10.1371/journal.pmed.1001308 22984354 PMC3439420

[19] Else H : Abstracts written by ChatGPT fool scientists. *Nature.* 2023;613(7944):423. 10.1038/d41586-023-00056-7 36635510

[20] Gao CA Howard FM Markov NS : Comparing scientific abstracts generated by ChatGPT to real abstracts with detectors and blinded human reviewers. *NPJ Digit. Med.* 2023;6(1):75. 10.1038/s41746-023-00819-6 37100871 PMC10133283

[21] Biswas S : ChatGPT and the Future of Medical Writing. *Radiology.* 2023;307(2):e223312. 10.1148/radiol.223312 36728748

[22] Salvagno M Taccone FS Gerli AG : Can artificial intelligence help for scientific writing? *Crit. Care.* 2023;27(1):75. 10.1186/s13054-023-04380-2 36841840 PMC9960412

[23] Korinek A : Language Models and Cognitive Automation for Economic Research. February 2023. 10.3386/w30957

[24] Stokel-Walker C Van Noorden R : What ChatGPT and generative AI mean for science. *Nature.* 2023;614(7947):214–216. 10.1038/d41586-023-00340-6 36747115

[25] Lund B Ting W : Chatting about ChatGPT: How May AI and GPT Impact Academia and Libraries? January 22, 2023. 10.2139/ssrn.4333415

[26] Liebrenz M Schleifer R Buadze A : Generating scholarly content with ChatGPT: ethical challenges for medical publishing. *Lancet Digit. Health.* 2023;5(3):e105–e106. 10.1016/S2589-7500(23)00019-5 36754725

[27] Hosseini M Horbach SPJM : Fighting reviewer fatigue or amplifying bias? Considerations and recommendations for use of ChatGPT and other Large Language Models in scholarly peer review. *Res. Sq.* February 20, 2023;rs.3.rs-2587766. 10.21203/rs.3.rs-2587766/v1 37198671 PMC10191680

[28] Robertson Z : GPT4 is Slightly Helpful for Peer-Review Assistance: A Pilot Study. June 16, 2023. 10.48550/arXiv.2307.05492

[29] Hosseini M Rasmussen LM Resnik DB : Using AI to write scholarly publications. *Account. Res.* 2024;31(7):715–723. 10.1080/08989621.2023.2168535 36697395 PMC10366336

[30] Chen Y Eger S : Transformers Go for the LOLs: Generating (Humourous) Titles from Scientific Abstracts End-to-End. December 26, 2023. 10.48550/arXiv.2212.10522

[31] Rehman T Sanyal DK Chattopadhyay S : Can pre-trained language models generate titles for research papers? October 13, 2024. 10.48550/arXiv.2409.14602

[32] Klein D : Implementing a General Framework for Assessing Interrater Agreement in Stata. *Stata J.* 2018;18(4):871–901. 10.1177/1536867X1801800408

[33] Gwet KL : *Handbook of Inter-Rater Reliability: The Definitive Guide to Measuring the Extent of Agreement among Raters.* Advances Analytics, LLC; Fourth ed. 2014.

[34] Sebo P Lucia S de : Performance of machine translators in translating French medical research abstracts to English: A comparative study of DeepL, Google Translate, and CUBBITT. *PloS One.* 2024;19(2):e0297183. 10.1371/journal.pone.0297183 38300946 PMC10833527

[35] Landis JR Koch GG : The measurement of observer agreement for categorical data. *Biometrics.* 1977;33(1):159–174. 10.2307/2529310 843571

[36] Weber-Wulff D Anohina-Naumeca A Bjelobaba S : Testing of detection tools for AI-generated text. *Int. J. Educ. Integr.* 2023;19(1):1–39. 10.1007/s40979-023-00146-z

[37] Walters WH : The Effectiveness of Software Designed to Detect AI-Generated Writing: A Comparison of 16 AI Text Detectors. *Open.* *Inf. Sci.* 2023;7(1). 10.1515/opis-2022-0158

[38] Clark E August T Serrano S : All That’s ‘Human’ Is Not Gold: Evaluating Human Evaluation of Generated Text. Zong C Xia F Li W , editors. *Proceedings of the 59th Annual Meeting of the Association for Computational Linguistics and the 11th International Joint Conference on Natural Language Processing (Volume 1: Long Papers).* Association for Computational Linguistics;2021;7282–7296. 10.18653/v1/2021.acl-long.565

[39] Else H : ‘Tortured phrases’ give away fabricated research papers. *Nature.* 2021;596(7872):328–329. 10.1038/d41586-021-02134-0 34354273

[40] AL-Rawas M Qader OAJA Othman NH : Identification of dental related ChatGPT generated abstracts by senior and young academicians versus artificial intelligence detectors and a similarity detector. *Sci. Rep.* 2025;15(1):11275. 10.1038/s41598-025-95387-y 40175423 PMC11965432

[41] Nabata KJ AlShehri Y Mashat A : Evaluating human ability to distinguish between ChatGPT-generated and original scientific abstracts. *Updat. Surg.* January 24, 2025;77:615–621. 10.1007/s13304-025-02106-3 39853655

[42] Caiado AJA Hahsler M : AI Content Self-Detection for Transformer-based Large Language Models. December 28, 2023. 10.48550/arXiv.2312.17289

[43] Science journals set new authorship guidelines for AI-generated text National Institute of Environmental Health Sciences; Accessed June 13, 2025. Reference Source

[44] Resnik DB Hosseini M : Disclosing artificial intelligence use in scientific research and publication: When should disclosure be mandatory, optional, or unnecessary? *Account. Res.* 1–13. 10.1080/08989621.2025.2481949 40126451 PMC12353913

[45] Lund BD Naheem KT : Can ChatGPT be an author? A study of artificial intelligence authorship policies in top academic journals. *Learn Publ.* 2024;37(1):13–21. 10.1002/leap.1582

[46] Lund B Ting W Mannuru NR : ChatGPT and a New Academic Reality: Artificial Intelligence-Written Research Papers and the Ethics of the Large Language Models in Scholarly Publishing. March 15, 2023. 10.2139/ssrn.4389887

[47] Sebo P Nie B Wang T : Can ChatGPT write better scientific titles? A comparative evaluation of human-written and AI-generated titles. *Open Science Framework.* 2025. 10.17605/OSF.IO/NF8ZR PMC12982995 41835981

