# Supplementary material for: Can ChatGPT write better scientific titles? A comparative evaluation of human-written and AI-generated titles
Source: F1000Res. 2026 Feb 5;14:1470. Originally published 2025 Dec 30. [Version 2] doi: 10.12688/f1000research.173647.2 (PMC12982995; doi:10.12688/f1000research.173647.2)
Supplement: Peer review history

# Reviewer report: Reviewer response for version 2

**DOI:** 10.5256/f1000research.196161.r455891

**Published:** 2026-03-25

**Authors:** Renz Alvin Gabay

**Affiliations:** 1 Sorsogon State University, Sorsogon, Philippines

No further comments.

Is the work clearly and accurately presented and does it cite the current literature?

Yes

If applicable, is the statistical analysis and its interpretation appropriate?

Partly

Are all the source data underlying the results available to ensure full reproducibility?

Yes

Is the study design appropriate and is the work technically sound?

Yes

Are the conclusions drawn adequately supported by the results?

Partly

Are sufficient details of methods and analysis provided to allow replication by others?

Yes

Reviewer Expertise:

Science Education, Physics Education, AI in Education

I confirm that I have read this submission and believe that I have an appropriate level of expertise to confirm that it is of an acceptable scientific standard.

# Reviewer report: Reviewer response for version 2

**DOI:** 10.5256/f1000research.196161.r455890

**Published:** 2026-03-12

**Authors:** Aaron A. Funa

**Affiliations:** 1 Sorsogon State University, Sorsogon, Philippines

The manuscript is timely and well executed overall, but several points still require clarification. First, the authors should clarify whether any co-authors were included among the 21 raters, as the initials and affiliations reported in Table 1 appear to overlap with the author list despite the Methods describing the raters as independent. Second, the statistical rationale for the preference model should be explained more clearly, particularly why the mixed-effects model appears to include a random intercept for rater but not article. Third, the conclusions should remain closely aligned with what was actually measured, namely perceived alignment with the abstract, appeal, and overall preference, rather than implying broader superiority of AI-generated titles. Finally, the comparison between AI-generated titles from 2025 and human-written titles from 2000 should be emphasized as a central interpretive limitation, since part of the observed advantage may reflect changing title conventions over time. Overall, these issues appear to require clarification rather than major redesign, and the study remains a well-conceived and valuable contribution.

Is the work clearly and accurately presented and does it cite the current literature?

Partly

If applicable, is the statistical analysis and its interpretation appropriate?

Partly

Are all the source data underlying the results available to ensure full reproducibility?

Yes

Is the study design appropriate and is the work technically sound?

Yes

Are the conclusions drawn adequately supported by the results?

Partly

Are sufficient details of methods and analysis provided to allow replication by others?

Yes

Reviewer Expertise:

STEM Education; Science Education; Nursing Education

I confirm that I have read this submission and believe that I have an appropriate level of expertise to confirm that it is of an acceptable scientific standard, however I have significant reservations, as outlined above.

# Reviewer report: Reviewer response for version 2

**DOI:** 10.5256/f1000research.196161.r458467

**Published:** 2026-03-12

**Authors:** Nurul Aini

**Affiliations:** 1 English Education, Faculty of Teacher and Training, UIN Syekh Wasil Kediri, Kediri, East Java, Indonesia

Feedbacks:

1. Since it focuses on academic writing (scientific) but in the scope of general internal medicine, in the introduction part, it is very urgent to activate the readers’ prior knowledge related to those 2 things.  Write sentences showing the information related to medical things to avoid ambiguity. Locus and scope of the research are essential to bring the readers’ prior knowledge to the same page. My first impression, reading the introduction part, is that I relate to academic writing in the education area. Explore more issues of academic writing in the medical area.
2. We randomly selected five articles published between January 1 and December 31, 2000. To minimize the subjectivity and raise the objectivity, give a critical argument of why selecting those articles in 2000 (although the limitation is already stated but highlighting the reasons makes the article more academically sound). Why not the latest updated article, considering the novelty?
3. Survey: give detailed information on the total number of items (how many items being asked). Was it self-made? Adopted or adapted from the expert? From what framework was the survey developed? Was there any pilot testing of the instrument before a real one? Did you use any validity and reliability or things such so? Also, where did you take the scaling criteria of 0-10 from (mention the framework/expert) to strengthen the theory.
4. Be consistent in using the term fifty articles and 50 articles (numbering and letter).
5. In addition, future work could examine how title preferences correlate with actual article impact, such as downloads, citations, or Altmetric scores, to determine whether rater judgments align with broader readership behavior. Another key area for future research is to understand the mechanisms behind rater preferences (in implication).

 Future research could expand upon this work by including more diverse raters, evaluating newer articles, testing various prompting strategies, and incorporating additional dimensions of title quality. Despite these limitations, our findings provide valuable insights into the potential of LLMs to assist in academic title generation and highlight the subjective nature of title preferences (in limitations).  Are they in line? Who not stating in one part by simplifying the statements as both are targeted to the future researchers.

Is the work clearly and accurately presented and does it cite the current literature?

Yes

If applicable, is the statistical analysis and its interpretation appropriate?

Yes

Are all the source data underlying the results available to ensure full reproducibility?

Yes

Is the study design appropriate and is the work technically sound?

Yes

Are the conclusions drawn adequately supported by the results?

Yes

Are sufficient details of methods and analysis provided to allow replication by others?

Partly

Reviewer Expertise:

Technology, Artificial Intelligence in Education,

I confirm that I have read this submission and believe that I have an appropriate level of expertise to confirm that it is of an acceptable scientific standard.

# Reviewer report: Reviewer response for version 2

**DOI:** 10.5256/f1000research.196161.r458463

**Published:** 2026-03-05

**Authors:** Yustinus Calvin Gai Mali

**Affiliations:** 1 Universitas Kristen Satya Wacana, Salatiga, Indonesia

This article is well written and presents an interesting discussion on AI's ability to generate journal article titles. To enhance the manuscript, the authors should clarify the importance of conducting this study in the introduction and include a paragraph outlining the practical benefits of the study, specifying who will benefit most. Moreover, the methods section needs clarification, including why the 2023 JCR was chosen over the most recent edition. To improve transparency, the authors could share samples of their ChatGPT interactions using the standardized prompt: write a title for this scientific article based on the abstract below. This would help readers learn from their approach and apply it in their research. For statistical analysis, the authors should cite one or two prior studies that used similar tests on comparable datasets. Finally, given the prevalence of AI, the authors should consider adding a Gen AI Statement to disclose any AI tools used to write or improve the grammar of their manuscript.

Is the work clearly and accurately presented and does it cite the current literature?

Yes

If applicable, is the statistical analysis and its interpretation appropriate?

I cannot comment. A qualified statistician is required.

Are all the source data underlying the results available to ensure full reproducibility?

Yes

Is the study design appropriate and is the work technically sound?

Partly

Are the conclusions drawn adequately supported by the results?

Yes

Are sufficient details of methods and analysis provided to allow replication by others?

Partly

Reviewer Expertise:

Educational Technology, English Education

I confirm that I have read this submission and believe that I have an appropriate level of expertise to confirm that it is of an acceptable scientific standard, however I have significant reservations, as outlined above.

# Reviewer report: Reviewer response for version 2

**DOI:** 10.5256/f1000research.196161.r458465

**Published:** 2026-03-04

**Authors:** Helmi Ben Saad

**Affiliations:** 1 Faculty of Medicine of Sousse, Université de Sousse, Sousse, Tunisia

I read with a great pleasure the interesting paper titled: Can ChatGPT write better scientific titles? A comparative evaluation of human-written and AI-generated titles. Below is my detailed peer-review report.

 1. Strengths

 The paper has some strengths. First, it is a timely and original research question. The study addresses an emerging and highly relevant issue in scientific publishing. Second, it has a rigor methodological (blinded paired comparison, randomized title order, mixed-effects modeling accounting for rater and article clustering, and appropriate use of ordinal logistic regression for bounded scales). Third, it used pre-AI articles (year 2000), which is a major strength, ensuring that original titles were not AI-assisted. Fourth, it opted for a Human-centered evaluation: unlike prior studies relying on automated metrics, this study evaluates human perception. Fifth, the authors opted for transparency (open data availability (OSF), clear description of statistical methods, and detailed reporting of inter-rater agreement).

 2. Limitations

 The paper has some limitations, which should be reported inside the paper. First, there is a temporal bias: titles from 2000 may not reflect current stylistic norms. AI models trained on contemporary data may therefore have an inherent stylistic advantage. Second, there is a small and geographically clustered rater sample: N = 21 is modest, majority f researchers were from China and Switzerland, and limited representation of broader editorial communities). Third, raters were non-specialist raters (raters were not necessarily domain experts for each article topic, potentially limiting nuanced assessment of title–abstract alignment). Sixth, there is a restricted outcome dimensions, since only perceived alignment, appeal, and preference were assessed. Important aspects were not evaluated such as epistemic caution, overstatement risk, precision of claims, indexing optimization, and ethical tone. Fifth, a single-model evaluation was used: only GPT-4.0 was tested. No comparison with other LLMs or prompt engineering strategies. Finally, there is a zero-shot prompting: While ecologically valid, it may underestimate the true potential of LLM-assisted titling.

 3. Title

 The current title ( *Can ChatGPT write better scientific titles? A comparative evaluation of human-written and AI-generated titles*) is clear, engaging, and accurately reflects the study’s central research question and comparative design. It appropriately highlights the intervention (ChatGPT), the outcome (quality of scientific titles), the comparator (human-written titles), and the study type (comparative evaluation). However, the term “better” is inherently subjective. Since the study evaluates *perceived alignment, appeal, and preference*. It may be preferable to reflect this more explicitly to avoid overgeneralization.

 I suggest this long and more precise title: Perceived Alignment, Appeal, and Preference of Artificial Intelligence Generated versus Human-Written Scientific Titles: A Blinded Comparative Study Using GPT-4.0

 4. Abstract

 The abstract is well structured (Background, Objective, Methods, Results, Conclusions). It adheres to conventional scientific reporting standards. It clearly states rationale and gap in the literature, describes study design, sampling frame, and statistical approach, reports key quantitative results (means, p-values, ORs). Its conclusions are generally aligned with findings. However, some points need improvement. First, the term “better” is implied in the results and conclusions but should be contextualized as *perceived superiority*. Second, clarify that “alignment” refers to *perceived* alignment rather than objective accuracy. Third, avoid abbreviations misuse: define AI, GPT, AC at first use.

 5. Keywords

 Opt for MeSH terms

 Classify keywords in alphabetical order

 Avoid citing as keywords terms used in the title or the abstract

 6. Ethical guidelines

 The manuscript states that: No personal health data were collected, ethics approval was not required under Swiss law (HRA art. 2), and raters were informed and participated voluntarily. This appears compliant with local legal requirements. However, the authors may consider: Explicitly stating whether institutional review board consultation was sought or formally waived, and clarifying whether deception (non-disclosure of AI generation) was ethically reviewed or justified.

 7. Figures and tables

- Figures: Can authors consider adding effect size visualization (e.g., forest plot of ORs).

Figures: Define all used abbreviations (ID AI ) at the bottom of the table.

  

- Tables: Minor suggestions: Table 2 may be excessively granular for main text; consider moving full version to Supplementary Material, Ensure consistent formatting of ORs and Cis, and Standardize decimal places throughout.

Tables: Define all used abbreviations (AI IQR SD OR CI) at the bottom of the table.

 8. References

- Some web-based references may require access dates standardized.
- Avid references resented as preprints and arXiv citations.
- Rectify references 23, 25, 28, 30, 31, 42, 43, 46, ???

9. Use of AI chatbots during preparation

 The manuscript evaluates ChatGPT but does not clearly state whether AI tools were used in writing the manuscript itself. Given the subject matter, transparency is essential.

 I recommend adding a dedicated statement related to the use of AI during the writing of the paper.

 10. Additional remarks and suggestions

 10.1. Conceptual clarification

 The manuscript carefully reframes “accuracy” as “perceived alignment.” This is appropriate. Continue emphasizing that this is a perceptual, not epistemic, superiority.

 10.2. Overinterpretation risk

 The conclusion should avoid implying that AI titles are objectively superior. Suggested wording: Replace “may enhance scientific communication” with “may enhance perceived clarity and appeal under specific conditions.”

 10.3. Statistical interpretation

 The OR of 4.4 for perceived alignment is substantial. Consider discussing: Practical magnitude, and whether effect sizes correspond to meaningful real-world differences.

 10.4. Potential biases

- Cultural differences in stylistic preferences may influence ratings.
- LLM training bias toward assertive phrasing could affect appeal ratings.

10.5. Writing and organization

- Slight reduction of repetition in discussion.
- Tightening of some longer paragraphs for readability.

10.6. Misuse of references           

 Several sentences are lacking references: in the introduction section add references after: communication (L1), for peer review (L2), is intense (L4), complex task, or appeal, readable, community, scientific writing, generation, Chen and Erger,

 10.7. Misuse of abbreviations

 Define abbreviations at first use: see ChatGPT in the introduction,

 10.8. Article structure

 Objectives must be reported at the end of the introduction not in the beginning of the methods section.

 The paragraph (in the results section) named “Perceived accuracy and appeal ratings” should be moved to the methods section.

 10.9. Sample size

 Authors are asked to calculate the sample size: why 21? Why not 10 or 100?

Is the work clearly and accurately presented and does it cite the current literature?

Yes

If applicable, is the statistical analysis and its interpretation appropriate?

Yes

Are all the source data underlying the results available to ensure full reproducibility?

Yes

Is the study design appropriate and is the work technically sound?

Yes

Are the conclusions drawn adequately supported by the results?

Partly

Are sufficient details of methods and analysis provided to allow replication by others?

Partly

Reviewer Expertise:

Medical writing, Use of AI, respiratory physiology

I confirm that I have read this submission and believe that I have an appropriate level of expertise to confirm that it is of an acceptable scientific standard, however I have significant reservations, as outlined above.

# Reviewer report: Reviewer response for version 1

**DOI:** 10.5256/f1000research.191483.r446770

**Published:** 2026-01-29

**Authors:** Renz Alvin Gabay

**Affiliations:** 1 Sorsogon State University, Sorsogon, Philippines

**Overall Assessment**

 This manuscript addresses a timely and relevant question: whether large language models (LLMs), specifically ChatGPT-4.0, can generate scientific titles that are perceived as more accurate, appealing, and preferable than human-written titles. The study is clearly written, methodologically transparent, and thoughtfully situated within the emerging literature on AI-assisted academic writing. The use of blinded human raters, articles published before the advent of generative AI, and multiple statistical approaches are notable strengths.

 At the same time, several aspects of the design, interpretation, and framing would benefit from clarification or refinement. Most of these do not undermine the core findings but would strengthen the rigor, scope, and interpretability of the study. With revisions, this work has strong potential to make a meaningful contribution to the literature on AI in scientific communication.

**1. Conceptualization of “Perceived Accuracy”**

 Strength:

 The authors appropriately focus on reader-centered evaluation rather than automated metrics, which is a valuable contribution compared to prior studies.

 Suggestion:

 The construct of “perceived accuracy” would benefit from clearer conceptual framing. Because raters evaluated titles based only on the abstract (and were not domain experts for each topic), the measure appears to capture *perceived alignment between title and abstract* rather than factual or methodological accuracy of the study itself.

- The authors may consider clarifying this distinction throughout the manuscript.
- Rephrasing some claims to emphasize *perceived representativeness* or *abstract–title alignment* would improve conceptual precision.

**2. Use of Articles from the Year 2000**

 Strength:

 Selecting pre-AI articles is an elegant design choice that convincingly eliminates the possibility of AI-assisted human titles.

 Suggestion:

 At the same time, this introduces a potential temporal effect: writing conventions, stylistic norms, and reader expectations may have changed over 25 years. AI-generated titles, trained on modern scientific language, may naturally align better with contemporary preferences.

- The authors acknowledge this as a limitation, but it may merit stronger emphasis in both the Discussion and Conclusion.
- Slightly tempering general claims (e.g., “AI-generated titles can surpass human-written titles”) to reflect this context would enhance interpretive balance.

**3. Scope and Generalizability**

 Strength:

 The study design is well-controlled within a clearly defined domain (high-impact general internal medicine journals), which enhances internal validity.

 Suggestion:

 Some discussion sections extend the implications to scientific communication broadly, including lower-tier journals and other disciplines.

- The authors might consider more explicitly limiting generalizations to similar contexts (e.g., biomedical research, non-specialist academic readers).
- Framing broader claims as hypotheses for future research would maintain scholarly caution while preserving the manuscript’s relevance.

**4. Interpretation of “Preference” as Title Quality**

 Strength:

 Using overall preference as an outcome is intuitive and directly relevant to how readers interact with scientific articles.

 Suggestion:

 Preference, appeal, and perceived accuracy are inherently subjective constructs and may not fully capture other dimensions of title quality, such as precision, cautiousness of claims, or indexing suitability.

- The Discussion could benefit from acknowledging that higher appeal does not always equate to higher epistemic rigor.
- A brief reflection on possible trade-offs (e.g., rhetorical optimization versus conservative scientific framing) would enrich the interpretive depth.

**5. Modeling of Ordinal Data**

 Strength:

 The authors appropriately use non-parametric tests for paired comparisons and apply multilevel models to account for clustering by rater.

 Suggestion:

 The use of negative binomial regression for 0–10 ordinal ratings could be more fully justified, as these scores are not count data in the conventional sense.

- Providing a short rationale for this choice, or referencing prior studies that have used similar approaches, would strengthen methodological transparency.
- Alternatively, mentioning that the main conclusions were consistent across analytic approaches (if true) would reassure readers.

**6. Inter-Rater Agreement**

 Strength:

 Reporting both percent agreement and Gwet’s AC with weighted coefficients is commendable and demonstrates careful attention to reliability.

 Suggestion:

 Given that agreement was in the moderate-to-substantial range, the manuscript might briefly note that judgments of title quality remain partly subjective.

- Framing the findings as reflecting *consistent tendencies rather than unanimous consensus* would appropriately contextualize the results.

**7. Ethics and Responsible Use of AI**

 Strength:

 The manuscript commendably addresses ethical issues such as transparency, authorship, and the potential homogenization of scientific writing.

 Suggestion:

 This section could be modestly expanded to engage more directly with current debates in scholarly publishing, such as:

- whether AI-assisted title generation constitutes authorship, editing, or technical assistance;
- how journals might operationalize disclosure of AI use.

This would strengthen the practical relevance of the study.

**8. Literature balance:**

 The Introduction provides a strong overview of recent studies on AI-assisted scientific writing, which effectively situates the work within the current technological discourse. To further strengthen the theoretical grounding, the authors may consider incorporating additional literature on scientific title construction, rhetorical framing, and scientometrics from information science and bibliometrics. Integrating these perspectives could enrich the conceptual framework and more firmly anchor the study in the broader scholarship on how titles function in academic communication.

Is the work clearly and accurately presented and does it cite the current literature?

Yes

If applicable, is the statistical analysis and its interpretation appropriate?

Partly

Are all the source data underlying the results available to ensure full reproducibility?

Yes

Is the study design appropriate and is the work technically sound?

Yes

Are the conclusions drawn adequately supported by the results?

Partly

Are sufficient details of methods and analysis provided to allow replication by others?

Yes

Reviewer Expertise:

Science Education, Physics Education, AI in Education

I confirm that I have read this submission and believe that I have an appropriate level of expertise to confirm that it is of an acceptable scientific standard, however I have significant reservations, as outlined above.

# Reviewer report: Reviewer response for version 1

**DOI:** 10.5256/f1000research.191483.r446771

**Published:** 2026-01-29

**Authors:** Aaron A. Funa

**Affiliations:** 1 Sorsogon State University, Sorsogon, Philippines

The study addresses a timely question and uses an appropriate blinded paired-title design: 50 abstracts from 10 high-impact general internal medicine journals (year 2000) were retitled with GPT-4.0, and 21 researchers rated each human vs AI title for accuracy and appeal (0–10) and chose an overall preference. The work is generally clear, technically sound, and supported by open underlying/extended data. However, two points must be fixed for scientific soundness: (1) correct the inconsistency in the reported rating scale (0–10 vs 1–10) and confirm the coding used in all analyses; and (2) reconsider modeling bounded ordinal ratings with negative binomial regression. An ordinal mixed-effects model (or a clearly justified alternative with sensitivity analyses) would better match the outcome type and should explicitly account for repeated measures across raters and articles. Additional improvements: specify the random article-selection procedure (sampling frame, method/seed) and standardize formatting (e.g., capitalization) for both title types to avoid confounding.

Is the work clearly and accurately presented and does it cite the current literature?

Partly

If applicable, is the statistical analysis and its interpretation appropriate?

Partly

Are all the source data underlying the results available to ensure full reproducibility?

Yes

Is the study design appropriate and is the work technically sound?

Yes

Are the conclusions drawn adequately supported by the results?

Partly

Are sufficient details of methods and analysis provided to allow replication by others?

Yes

Reviewer Expertise:

STEM Education; Science Education; Nursing Education

I confirm that I have read this submission and believe that I have an appropriate level of expertise to confirm that it is of an acceptable scientific standard, however I have significant reservations, as outlined above.
